# Whole mitogenomes reveal that NW Africa has acted both as a source and a destination for multiple human movements

**DOI:** 10.1038/s41598-023-37549-4

**Published:** 2023-06-27

**Authors:** Julen Aizpurua-Iraola, Amine Abdeli, Traki Benhassine, Francesc Calafell, David Comas

**Affiliations:** 1 Departament de Medicina i Ciències de la Vida, Institut de Biologia Evolutiva (CSIC-UPF), Universitat Pompeu Fabra, Barcelona, Spain; 2 Laboratorie de Biologie Cellulaire et Moléculaire, Faculté des Sciences Biologiques, Université des Sciences et de la Technologie Houari Boumediene, Alger, Algeria

**Keywords:** Biological anthropology, Genetic variation

## Abstract

Despite being enclosed between the Mediterranean Sea and the Sahara Desert, North Africa has been the scenario of multiple human migrations that have shaped the genetic structure of its present-day populations. Despite its richness, North Africa remains underrepresented in genomic studies. To overcome this, we have sequenced and analyzed 264 mitogenomes from the Algerian Chaoui-speaking Imazighen (a.k.a. *Berbers*) living in the Aurès region. The maternal genetic composition of the Aurès is similar to Arab populations in the region, dominated by West Eurasian lineages with a moderate presence of M1/U6 North African and L sub-Saharan lineages. When focusing on the time and geographic origin of the North African specific clades within the non-autochthonous haplogroups, different geographical neighboring regions contributed to the North African maternal gene pool during time periods that could be attributed to previously suggested admixture events in the region, since Paleolithic times to recent historical movements such as the Arabization. We have also observed the role of North Africa as a source of geneflow mainly in Southern European regions since Neolithic times. Finally, the present work constitutes an effort to increase the representation of North African populations in genetic databases, which is key to understand their history.

## Introduction

Modern humans have inhabited North Africa at least since 300,000 years ago^1^, and due to its geographic location, it has been home to many different populations and has seen multiple human movements throughout history^2^. However, knowledge about North African prehistory remains sketchy. From Morocco to Egypt and as far south as the Sahel, many and extremely diverse lithic industries dating to between 190 kya (thousand years ago) and 57 kya have been discovered and linked to the Aterian culture^3^. In Mesolithic times, two distinct lithic cultures are recognized in the Maghreb: the Iberomaurusian (~ 22–9 kya)^4^ and the Capsian (~ 10–6 kya)^5^, who are believed to have originated from the Paleolithic Aterian people^3^. Subsequently, the diffusion of the Neolithic (starting at ~ 5.5 kya) involved population growth, with diversified subsistence systems and an increase in sedentarism. These populations possibly gave rise to present day Amazigh (sing.)/Imazighen (pl.) populations, which are also known as *Berbers*^4,5^.

Direct genetic analysis of prehistoric samples in North Africa has been hampered by the harsh climatic conditions that resulted in poor DNA preservation. Nevertheless, the analysis of the oldest aDNA samples from the entire African continent comes from the Taforalt Iberomaurusian settlement in Morocco from ~ 15 kya. Their study revealed the affinity of Epipaleolithic North Africans with Epipaleolithic Near Eastern populations^6^. Additionally, a sub-Saharan component was also detected, although none of the present day or ancient Holocene African groups were found to be a good proxy for the source of this component. Posterior ancient and modern samples revealed a certain degree of genetic continuity in the region form the Later Stone Age to Neolithic populations in the Maghreb and evidenced a remarkable impact of the Neolithic transition coming from Europe probably via the strait of Gibraltar^7,8^. After the Neolithic era, the major demographic movements in the region were: (i) the trans-Saharan gene flow caused mainly due to the slave-trade starting during the Roman empire rule (first century BCE) through the Arab conquest until the nineteenth century and (ii) the Arabization^9^, starting in the seventh century CE and introducing gene flow from the Middle East and leading to an East to West genetic cline of the Middle Eastern component in North Africa^10^. Other possible external contributors were the Phoenicians, Romans, Vandals, Byzantines, Ottoman Turks and other Mediterranean populations.

Mitochondrial DNA (mtDNA) has been a widely used genetic marker in population genetics^11^ and North Africa is no exception. Most of the studies conducted have focused on either control region lineage frequencies in different populations^12–14^ or on particular lineages present in North African population^15–19^. Control region studies reported a high heterogeneity in the haplogroup distribution of North African populations^12,20^, with some East–West clinal distributions for some lineages. Haplogroups H, HV0, L1b, L3b and U6 are more frequent in Western North Africa; while M1, L0a, R0a, N1b, I and J are more frequent in Eastern North Africa^16,18^. Overall, the North African maternal genetic landscape can be divided in (i) West Eurasian origin lineages, which generally make up for the majority of the maternal pool in North African populations; (ii) the U6 and M1 autochthonous North African haplogroups, which have been observed at least since the Epipaleolithic era in the region^6^; and (iii) sub-Saharan L lineages^13^. This lineage diversity evidences the importance of North Africa as a scenario of different human migrations, the study of which has been the objective of different lineage-specific analyses. For instance, an ancient link with Europeans was inferred since Iberian post-Glacial expansion lineages (namely U5b1b, H1, H3 and V) were detected in Amazigh populations^16^ and the analysis of complete mitogenomes provided enough resolution to discover novel North African specific clades within these haplogroups with coalescence times of around 4 to 7 kya^15^, whose origin could be attributed to the Neolithic expansion from the Iberian Peninsula. The expansion of some of these European lineages did not stop in North Africa and reached sub-Saharan African populations^21^. Other studies have expanded on the link with sub-Saharan populations (although mainly through the L haplogroup mtDNAs present in Europe) and discovered both ancient and recent origin mitochondrial lineages in Europe and North Africa^12,19^. In agreement with this, North African U6 and M1 lineages have also been observed in Europe^14,17^. All these results indicate that humans have permeated the Sahara Desert and Mediterranean Sea barriers enabling gene flow during different periods.

In this context, Algeria, the largest country in Africa with an area of 2.4 million km^2^, is home to different linguistic groups that include not only Arab speakers (who mainly inhabit the cosmopolitan cities), but also to many different Tamazight or Berber speaking groups like the Chaoui or Shawiya, Kabyle, Mozabite, Zenate, Chleuh, or Touareg. Previous studies have highlighted the heterogeneity between different Algerian groups, which appeared not to be correlated with linguistic or geography^20^. However, these studies analyzed control region data, which due to its limited phylogeographic resolution when comparing with complete mitogenomes, did not provide fine-grained phylogeographic information on the origin of the lineages. In addition, the informativeness of the mitochondrial phylogenies depends directly on the amount of available data from reference populations. In this sense, North Africa has been traditionally underrepresented in population genetic which is exemplified by the presence of just a single North African population (the Mozabite) in a global genomic database like the Human Genome Diversity Project^22^ and the 4 samples (2 Mozabite and 2 from the Western Saharawi population) in the Simons Genome Diversity Project^23^.

In the present study we aim to contribute to the representation of genetically neglected regions such as mainland North Africa, where, to the best of our knowledge, no population-based complete mitogenome studies have been conducted so far. To that effect, we have analyzed 264 mitogenomes of individuals from the Chaoui Amazigh population. The Chaoui Imazighen inhabit the Aurès mountainous region in North Eastern Algeria, and despite being one of the largest Amazigh populations in North Africa, just two genetic studies analyzing Y-chromosome and autosomal STRs have covered this population^24,25^. We focus on the mtDNA diversity observed in three different localities in the Aurès region and analyze the time and geographic origin of the maternal lineages in our dataset.

## Materials and methods

### Samples and sequencing

**Figure 1:**
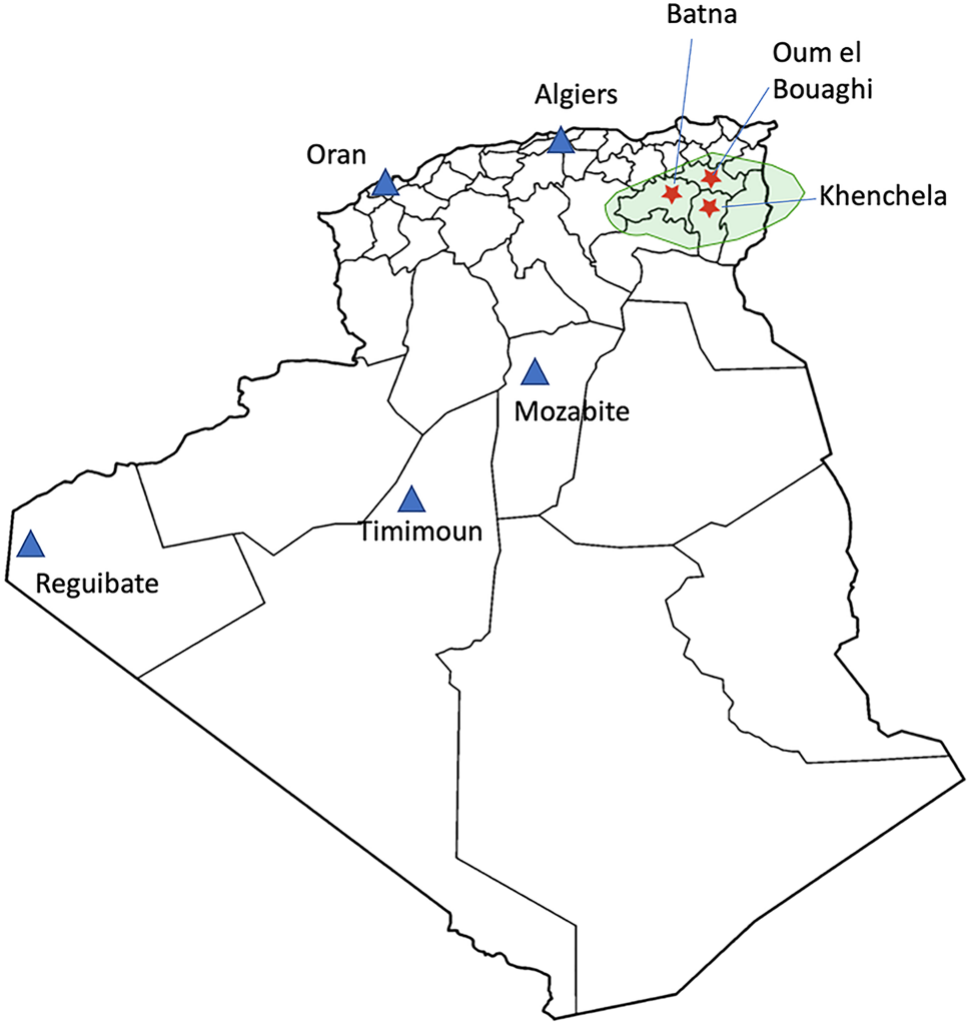
Map of Algeria with the three sampling sites in the Aurès marked with red stars. The green area represents the Aurès mountainous region. The map was created with the R package ‘rnaturalearth’^59^.

We amplified and sequenced the whole mtDNA from blood samples donated by 302 volunteers from three different localities in the Aurès region in Algeria: Batna (n = 136), Khenchela (n = 82) and Oum El Bouaghi (n = 84) (Fig. 1). All samples correspond to *Shawiya* or *Chaoui* speakers with all four grandparents from the Aurès region. A questionnaire and a written informed consent was obtained from the volunteers; the study complies with the ethical rules of all the institutions involved and has been approved by the *Laboratoire de Biologie Cellulaire et Moléculaire* at the *Faculté des Sciences Biologies* of Université de Sciences et Technologie Houari Boumedienne in Algiers and the CEIm-PSMAR IRB in Barcelona (2019/8900/I). All methods in this study were performed following the standard guidelines and regulations in accordance with the Declaration of Helsinki.

DNA was extracted using the QIAamp DNA blood mini kit (Qiagen GmbH, Hilden, Germany) following the manufacturer’s recommendations and was quantified using Quantifiler™ Human DNA Kit on 7500 SDS Reak-Time PCR System (Applied Biosystems). PCR amplifications were performed in four different fragments as in Ref.^26^. Nextera XT libraries were prepared and sequenced in a Miseq sequencer (Illumina) following the Illumina mtDNA Genome Guidelines^27^.

### Sequence processing

Sequences were processed according to the GATK best practices protocol^28^. First, an initial quality check was performed using FastQC^29^. Then, the raw sequencing reads were mapped against the revised Cambridge Reference Sequence (rCRS)^30^ using the BWA-MEM algorithm^31^. The PCR duplicates were removed with Picard tools^32^, base quality scores were recalibrated with GATK’s Base Quality Score Recalibration (BQSR)^33^, and a final quality report was obtained with Qualimap2^34^. Finally, the sequence variants were called with GATK tools HaplotypeCaller and GenotypeGVCFs^33^.

As a quality filter, we first set mean coverage values of above 15× for each of the amplified fragments and a Haplogrep quality score above 85%. Samples failing any these conditions were discarded unless they had at least a coverage of 5× across all the reference. 24 samples failed both the 15× coverage and the > 85% Haplogrep quality score, 7 samples failed the coverage filter and were discarded, and 8 samples failed just the Haplogrep score requirement, of which 4 of them were discarded since they contained regions with < 5× of coverage. Finally, three additional samples were discarded since we detected three pairs of third-degree relatives in our dataset after an autosomal genomewide kinship analysis with King software^35^. We ended up with a final number of 264 sequences (Batna n = 133, Khenchela n = 75, Oum El Bouaghi n = 56).

### Statistical analysis

Haplogroups were determined with Haplogrep v.2.4.0 using the forensic update of phylotree v.17^36^. Molecular diversity and summary statistics were calculated with the *pegas* package in R^37^ and AMOVA and Φst distances were computed with *poppr*^38^ and *ade4*^39^*.*

### Population analysis

Given the absence of whole mtDNA population reference datasets from North Africa, we downloaded control region data from different Algerian populations: Mozabite (n = 85)^40^, Timimoun (n = 73)^20^, Reguibate (n = 108)^20^, Oran (n = 333)^20,41^ and Algiers (n = 62)^20^.

### Phylogeographic analysis

We first selected the haplogroups with a count number of n ≥ 2 and blasted the mitogenomes within those haplogroups in Genbank searching for the most similar publicly available sequences. We downloaded the 20 most similar sequences based on the identity percentage obtained and built phylogenetic networks looking for North African specific clades. We define North African specific clades as the groups of sequences found in North Africa that belong to the same haplogroup and share at least one mutation (not accounting for the highly recurrent mutations listed in Ref.^42^) that separates them from other sequences. For each North African clade, we dated the internal Time to the Most Recent Common Ancestor (TMRCA) in addition to the time to the closest non-North African individual using BEAST 1.10^43^. For the case of sequences belonging to M1 and U6 haplogroups, we inferred (i) the coalescent time for each of the non-North African clades found within the sequences obtained from GenBank, and (ii) the coalescence time between each non-North African sequence (not forming clades) and the closest North African sequence.

For the BEAST analysis, we used a prior mutation rate of 2.355 × 10^–8^ substitutions per nucleotide per year, taking into account purifying selection as in Ref.^44^, the substitution model we used was the GTR as indicated by jModelTest2^45^ and set a strict clock model assuming all tree branches evolve at the same rate. We used UPGMA as the starting trees for the analysis, which was conducted in five independent runs with 15,000,000 iterations, sampling the result every 15,000 iterations. The resulting log files were combined using LogCombiner and the combined log file was checked with Tracer 1.7^46^ to ensure effective sample size (ESS) values over 200 for every parameter.

## Results

### mtDNA diversity in the Aurès region within the Algerian context

We generated a total of 264 complete mitogenomes with a mean coverage of 593.5× (Suppl. Figs. 1, 2) from three different localities in the Aurès mountainous region in North Eastern Algeria: Batna (n = 133), Khenchela (n = 75) and Oum El Bouaghi (n = 56) (Suppl. Table 1). We computed diversity statitics for the three localities (Suppl. Table 2A). Batna shows a slightly lower haplotype diversity in comparison to the other populations. We computed an AMOVA test to verify whether there was significant internal variation among the three localities, yielding a small but significant fraction of variance found among the localities (0.92%, p = 0.008), with Khenchela showing the highest *φ*_st_ values (Suppl. Table 2B).

**Figure 2:**
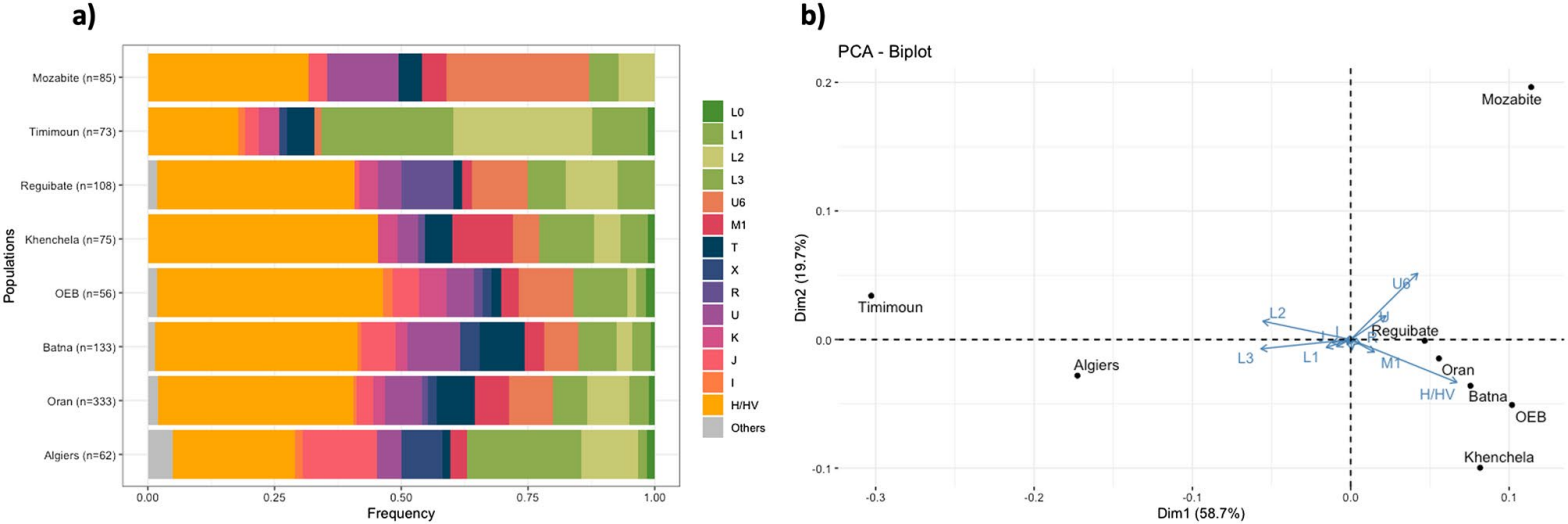
(**a**) The haplogroup composition for each of the Algerian populations based on HVS-I data and (**b**) principal component analysis (PCA) based on the haplogroup frequencies obtained from HVS-I data from Algerian populations.

Due to the lack of whole mtDNA population data from North African populations, we built a dataset of control region sequences from different parts of Algeria (see “Materials and methods” section). Within the Algerian context, we observe a clear heterogeneity in the haplogroup composition between the different populations as previously reported^20^ (Fig. 2a, Suppl. Table 3). The mtDNA haplogroup composition from the Aurès region shows high frequencies of West Eurasian (WE) superhaplogroups (mainly H/HV) with 60% of WE haplogroups in Khenchela and 74.4% and 69.6% for Batna and Oum El Bouaghi respectively (Fig. 1). Overall, Khenchela is also differentiated because of higher proportions of U6 and specially M1 North African sequences, being the locality with the highest frequency of M1 lineages (12%) among all the Algerian populations we considered. Regarding sub-Saharan lineages, Khenchela also showed the highest proportion of L haplogroup sequences among the Aurès region but well below the Amazigh population in Timimoun or the Arab population in Algiers.

Both haplogroup frequency-based distances and *φ*_st_ distances show the populations from the Aurès region are closer to the population in Oran and the Reguibate population (Fig. 2b, Suppl. Fig. 3). Khenchela shows higher *φ*_st_ distances (Suppl. Table 4) and also appears slightly separated from Batna and Oum El Bouaghi the haplogroup frequency-based PCA, probably due to the high M1 proportions.

### Origins of the Aurès mitogenome lineages

**Figure 3:**
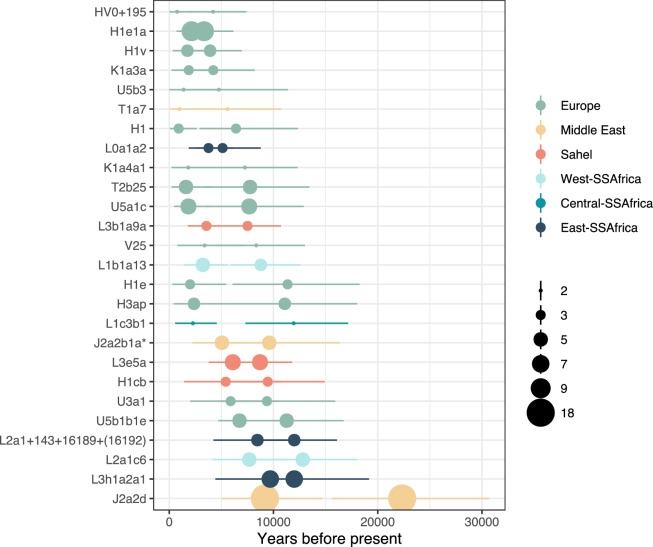
Inferred coalescent dates for all North African specific clades (left point) and inferred date to the closest non-North African sample (right point). Colour indicates the geographic origin and the point size refers to the number of sequences within the North African clades.

After exploring the phylogeographic origin of the lineages of non-North African origin in the Aurès sample, we found that 73 sequences from the Aurès region belonged to 26 different North African specific clades with origins outside Northern Africa (Fig. 3) (without accounting for the 18 clades formed by 31 identical sequences (Suppl. Fig. 26)). That is, the closest sequences to these clades had been sampled outside of North Africa, particularly in Europe, the Middle East, the Sahel, and West, Central, and East Africa.

Many European lineages that are present in the Aurès sample (i.e.: H1e1a, H1v, K1a3a, U5a1c, etc.) have internal coalescence ages of around 2000 years before present (ybp) and some are not only found in the Aurès region but also in other North African populations including the Canary Islands (Suppl. Figs. 4–15). Coalescence ages to the closest non-North African individuals, which serve as an upper limit of the origin of the lineage in North Africa, are more heterogeneous (ranging from ~ 3000 to ~ 11,000 ybp) as they are related to each haplogroup degree of representation in the literature. Besides, one lineage belonging to haplogroup U3a1c has an internal coalescence age of 5888 ybp (Fig. 3) suggesting European influence in the region dating as far back as ~ 6000 ybp.

Regarding Middle Eastern lineages, we found three North African specific clades also present in the Aurès region belonging to haplogroups T1a7, J2a2b1a* (sensu^47^) and J2a2d. The North African specific clade within T1a7 is just formed by two sequences from the Aurès and have a recent (967 ybp) coalescence age, while the two other clades are present in other North African populations (including the Canary Islands) and have prehistoric coalescence ages (5058 and 9179 ybp for J2a2b1a* and J2a2d respectively) (Fig. 3, Suppl. Figs. 16–18).

We also found an influence of populations from the Sahel region (mainly Fulani populations from Burkina Faso, Chad, Niger and Mali) as we found North African lineages within haplogroups H1cb, L3e5a and L3b1a9 with a putative origin in the Sahel region (Suppl. Figs. 19–21). The time origin of these lineages in North Africa appears to be prehistoric since all but one internal coalescence ages inferred are above 5000 ybp (Fig. 3). The branching pattern of the H1cb lineages shows the presence of a more basal sequence in Mauritania, while the Aurès specific clade stems from the Sahelian diversity, which could point to a “back to North Africa” migration of this lineage^21^. When considering the origin of the Sahelian L3 lineages in North Africa, we observe a number of different North African sequences outside the North African specific clades which might reflect independent migrations events throughout history.

Finally, we also found North African specific clades with Eastern, Western and Central sub-Saharan African origins. The time origin of these lineages is overall heterogenous with internal coalescent date inferences ranging from 2275 to 9696 ybp (Fig. 3). As in the case of the lineages with Sahelian origin, we observe many North African individuals with haplogroups from different regions of sub-Saharan Africa outside North African specific clades, which might also suggest independent migration events (Suppl. Figs. 22–25).

Although most North African clades were composed of non-clonal sequences separated by greater or less divergence time, we also observed North African specific clades belonging to non-North African haplogroups constituted by identical sequences, which could be a sign of very recent migration events into the region (Suppl. Fig. 26). The origin of most of these lineages seems to be European, although we find some Middle Eastern lineages like R0a2 which is present in Bedouins (Suppl. Fig. 27). Interestingly, two samples from the Aurès region share their haplotype with an ancient Phoenician sample collected in Sardinia belonging to the W5 haplogroup (Suppl. Fig. 28).

### Influence of the Aurès and North Africa over other territories

Besides the phylogeographic structure of non-North African sequences in Northern Africa we also analyzed the M1 and U6 lineages present in the Aurès region outside North Africa. These haplogroups originated, or at least greatly expanded, in North Africa^10,17,18^. For this purpose, we inferred the coalescence age between the closest non-North African and North African individuals belonging to haplogroups present in the Aurès sample. The results show that the time divergences of M1 and U6 lineages between North African and non-North African individuals are roughly uninterrupted along time and that most of these lineages are found in Southern Europe (Suppl. Fig. 29).

We found some European specific U6 and M1 clades for which we inferred the divergence time and found that many of the TMRCA of these clades fall between the present time and 5,000 ybp. Besides, these lineages are also found in Southern Europe (Suppl. Fig. 30).

## Discussion

Despite its extensive geographic range and the presence of numerous distinct populations, North Africa has generally received little attention in genetic studies. Algeria, the largest country in Africa, is home to several different linguistic groups including Arab speakers, who make up the majority in urban areas, and also several distinct Tamazight speaking groups, such as the Chaoui or Shawiya, Kabyle, Mozabite, Zenate, Chleuh, and Touareg. The genetic studies covering these populations, however, are still scarce. We present 264 new complete mitochondrial sequences from the Chaoui Amazigh group collected in three different localities in the Aurès region in North-eastern Algeria.

We observed small but significant variation among the three localities sampled in the Aurès region (0.92%, p = 0.008), with Khenchela being the locality contributing most to this local substructure. Looking at the linage composition of each of the sampling sites, Khenchela’s contribution to the local maternal substructure observed is possibly caused by the high proportion of M1 lineages, the highest among the Algerian populations examined in this study.

In an Algerian context, we analysed the HVS-I control region of different Algerian groups together with our samples and observed the genetic heterogeneity previously described in the country as well as for other Imazighen^9,12,20^. The haplogroup composition of the Aurès sample is close to that of the city of Oran (Fig. 2), the second largest city in Algeria and with an Arab majority, which might suggest genetic similarities between the Aurès and inhabitants from cosmopolitan cities. Their haplogroup composition is mainly composed by H/HV lineages evidencing the European influence in North Africa, although other West Eurasian haplogroups can also be observed in most Algerian populations in lower frequencies. Besides, we also observe a moderate frequency of M1 and U6 North African autochthonous lineages that have been present in the region since Palaeolithic times^6^, and finally sub-Saharan L lineages at frequencies ~ 20%.

Genetic and archaeological evidence have dated the first Western European influence in North Africa around 6000 ybp coming from the Iberian Peninsula and have linked it to the Neolithization process in North Africa^7,48^. In accordance with this, several European mitochondrial lineages discovered in the Fulani and other Sahelian populations (namely H1cb1 and U5b1b1b) have been linked to this same migration across the Gibraltar strait to North Africa from where it would have spread southwards to the Sahel^21,49,50^. After inspecting the time and geographic origin of the lineages present in the Aurès region and other parts of North Africa, we observed not only clades belonging to these specific haplogroups (H1cb1) but also other clades of West Eurasian origin with coalescent dates around the same period (U3a1c, ~ 6000 ybp and U5b1b1e, ~ 6700 ybp) that might also be linked to the migrations across the Gibraltar strait during the Early Neolithic period. The rest of European origin lineages found in the Aurès region and in other North African samples have more recent coalescent dates (between ~ 2000 and ~ 3000 ybp) indicating an entrance during historical times during the Phoenician, Roman or other contacts with Mediterranean populations. Nevertheless, given that some of these lineages are also present in the Canary Islands (H1e1a)^51^, their coalescence age should be at least higher than ~ 2000 ybp (the estimated age for the colonization of the archipelago^52^).

Regarding Middle Eastern origin lineages in North Africa, we have observed the presence of a North African specific J2a2d haplogroup with an estimated coalescence age of ~ 9100 ybp. This date predates the Neolithization period in North Africa and, given the link between the Taforalt samples and the Epipaleolithic Middle Eastern populations (Natufians)^6^, it could represent pre-agricultural contact connection between the Near East and North Africa, as it occurs with the Y-chromosome lineage E1b1b1a1 (M-78), which is very frequent in North Africa and is very closely related to the lineage E1b1b1b (M-123) found in Natufians and Pre-Pottery Neolithic Levantines^53^. The North African haplogroup J2a2b1a* (sensu^47^) also presents a prehistoric coalescent date around 5,000 ybp but its more recent origin could also be related to posterior migrations. The clade within T1a7 seems to be the only recent Middle Eastern lineage found in the Aurès region with a coalescence age of < 1000 ybp, and possibly reflects the incoming maternal lineages due to the Arabization or the Bedouin expansion^8,54^.

Finally, the genetic influence of sub-Saharan population in North Africa can be dated back to Epipaleolithic times, as the Taforalt individuals present a sub-Saharan component making up to approximately a third of their genomes^6^. Besides, prehistoric contacts between both sides of the Sahara have been mainly evidenced by mtDNA studies, since most recent genome wide studies have mostly been able to detect recent sub-Saharan admixture in North Africa driven by the extensive slave trade^55^. European haplogroups related to the Neolithic expansion (H1cb1 and U5b1b1b) have been observed in the Sahelian populations^21,49,50^ and at the same time, sub-Saharan lineages with high frequencies in Lake Chad Basin populations (L3e5) have been reported in North African populations and are believed to have a prehistorical origin^12,56^. We have detected the presence of these lineages in North Africa—in addition to another lineage with a presumed Sahelian origin (L3b1a9a)—and the coalescent times of these lineages agree with those inferred in previous studies pointing out to a trans-Saharan during the Green Sahara period (~ 10,000–5000 ybp)^56,57^. Besides, we have observed North African specific clades with even older coalescence ages (ranging from ~ 7600 to ~ 9700 ybp), whose origins seem less restricted to a particular region, and these could have also arrived at North Africa during the Green Sahara period. Finally, it is worth noting that the great majority of sub-Saharan sequences in North Africa do not form phylogenetic clades, and therefore, show no signals of having evolved in North Africa. This observation is compatible with a recent arrival of these sequences to the region as suggested by recent genomewide evidence^9,55^.

Our results evidence that North Africa has not only acted as a sink of different human migrations, but also as a source and as a bidirectional corridor. North Africa’s role as a corridor is evidenced by the European lineages mentioned earlier that crossed the Gibraltar strait and the Sahara and are nowadays present in both North African and sub-Saharan populations. Our results, in agreement with previous studies^19^, also indicate that some lineages took the opposite direction. In fact, most of the North African clades with sub-Saharan origin contain sequences sampled in Europe (Suppl. Figs. 21–24). Finally, North Africa’s role as a source is evidenced by the presence of the autochthonous M1 and U6 sequences outside North Africa. Our results, despite focusing just on lineages present in the Aurès region, point to a roughly uninterrupted geneflow from North Africa mainly towards Southern Europe, since at least ~ 5000 ybp as suggested by the coalescence ages inferred from most of the European specific U6 and M1 clades observed. This agrees with previous results that point out higher historic spreads of North African lineages possibly during Phoenician rule, the Roman empire or the Arab expansion^19^.

This present work, despite the multiple studies focused on specific haplogroups within North Africa^15,17,18,21,58^, constitutes the first population-based whole mitogenome study of a mainland North African population. This, although it represents a positive effort to increase the representation of undersampled populations in public databases and in the literature, can also give rise to potential limitations. Due to the limited number of publicly available mitochondrial genomes from sub-Saharan Africa and North Africa in comparison with Europe, we need to be cautious when interpreting these results, since African populations remain largely uncharacterized from a population genetics standpoint and some of the phylogeographic inferences might change with the addition of more genetic data.

## Supplementary Information

Supplementary Tables.

Supplementary Figures.

## Data Availability

The mitochondrial genomes produced in this study are available on GenBank with the accession numbers: OQ884717–OQ884980.
