# Supplementary material for: Whole mitogenomes reveal that NW Africa has acted both as a source and a destination for multiple human movements
Source: Sci Rep. 2023 Jun 27;13:10395. doi: 10.1038/s41598-023-37549-4 (PMC10300034; doi:10.1038/s41598-023-37549-4)
Supplement: Supplementary file 2 — Supplementary Figures. [file 41598_2023_37549_MOESM2_ESM.pdf]

Supplementary Material

Whole mitogenomes reveal that NW Africa has acted both as a source and a destination for multiple human movements.

Julen Aizpurua-Iraola[1], Amine Abdeli[2], Traki Benhassine[2], Francesc Calafell[1], David Comas[1]

1. Institut de Biologia Evolutiva (CSIC-UPF), Universitat Pompeu Fabra, Departament de Medicina i Ciències de la Vida, Barcelona, Spain
2. Laboratorie de Biologie Cellulaire et Moléculaire, Faculté des Sciences Biologiques, Université des Sciences et de la Technologie Houari Boumediene, Alger, Algeria

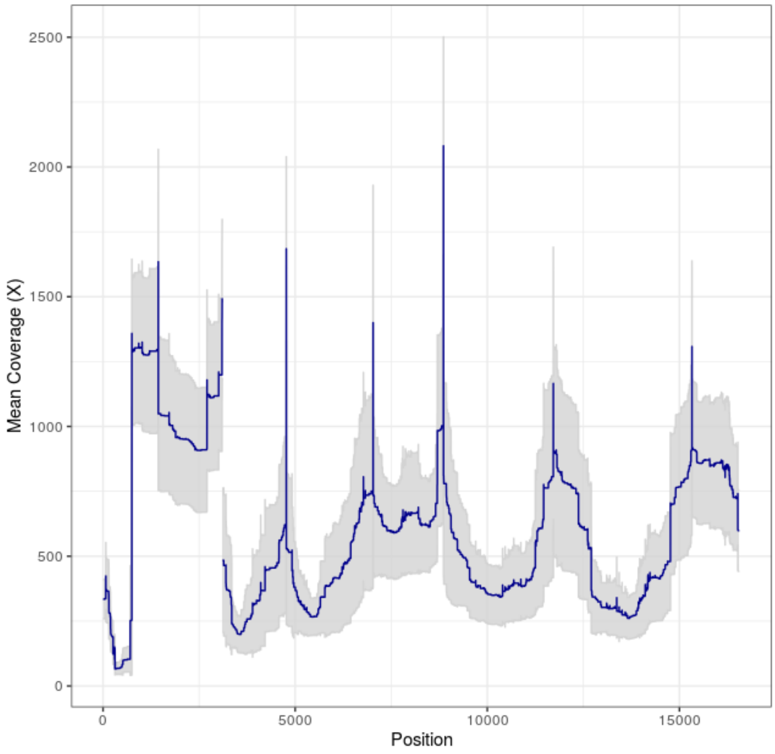

Figure 1 Mean coverage (blue line) and standard deviation (gray shadow) along the mtDNA reference sequence.

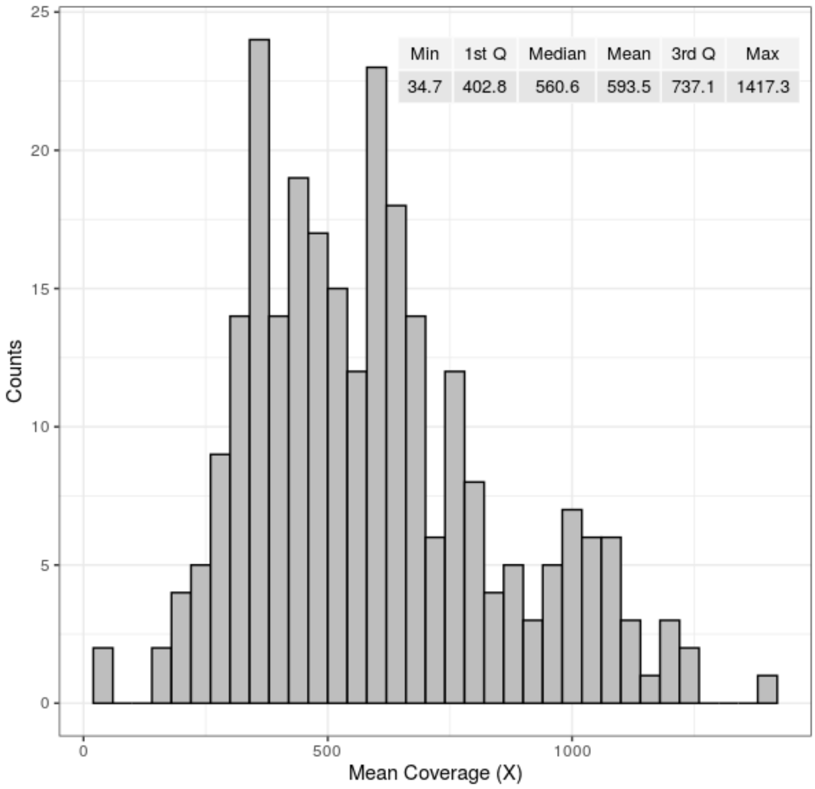

Figure 2 Distribution of mean coverage per sample.

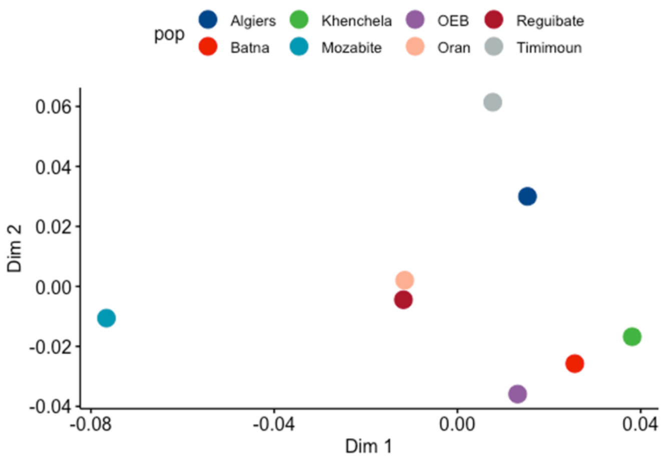

Figure 3 Multidimensional Scaling analysis of the population pairwise φ_{ST} distances based on the HVS-I region data from Algerian populations.

European Lineages in the Aurès population:

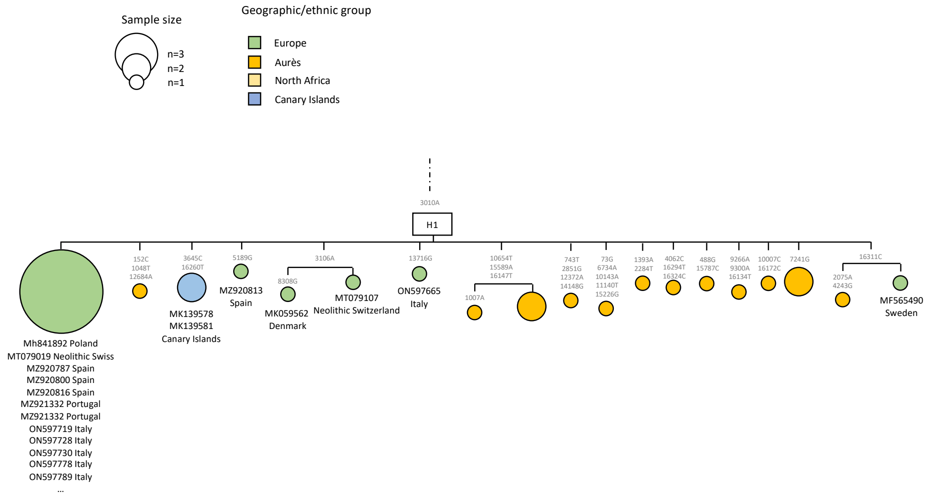

Figure 4 Maximum parsimony tree of the H1 haplogroup mitogenomes in the Aurès population. The mtDNA variants are indicated along the branches of the tree.

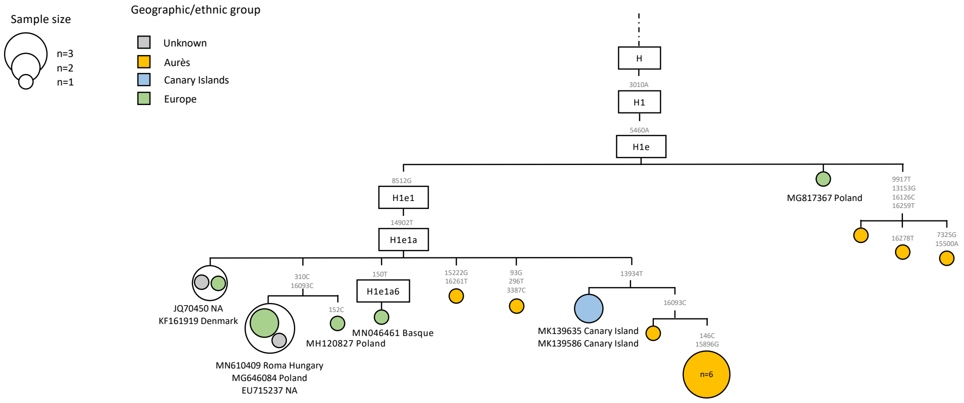

Figure 5 Maximum parsimony tree of the H1e and H1e1 haplogroup mitogenomes in the Aurès population. The mtDNA variants are indicated along the branches of the tree.

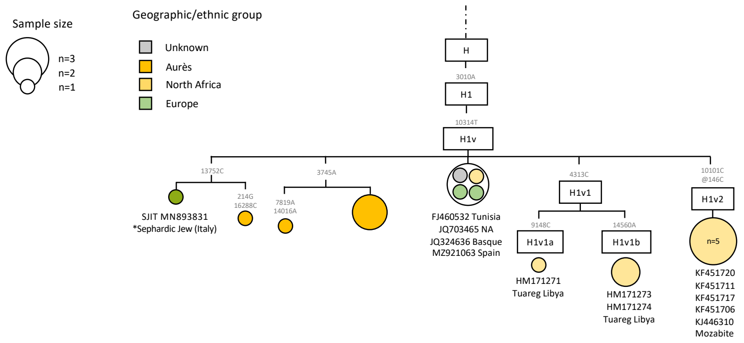

Figure 6 Maximum parsimony tree of the H1v haplogroup mitogenomes in the Aurès population. The mtDNA variants are indicated along the branches of the tree.

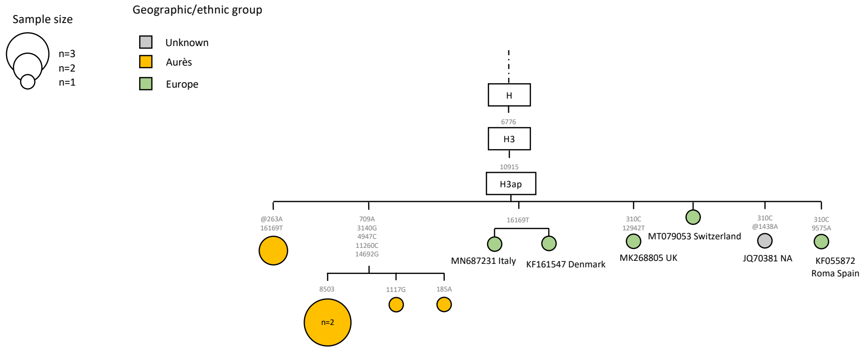

Figure 7 Maximum parsimony tree of the H3ap haplogroup mitogenomes in the Aurès population. The mtDNA variants are indicated along the branches of the tree.

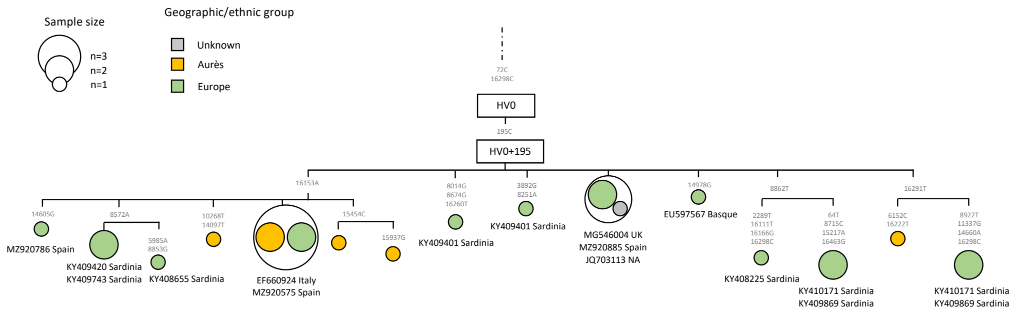

Figure 8 Maximum parsimony tree of the HV0+195 haplogroup mitogenomes in the Aurès population. The mtDNA variants are indicated along the branches of the tree.

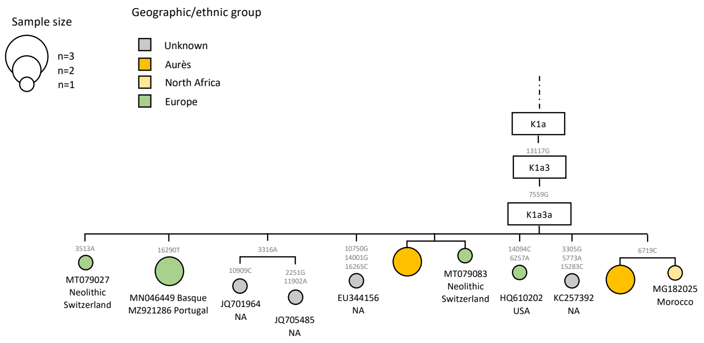

Figure 9 Maximum parsimony tree of the K1a3a haplogroup mitogenomes in the Aurès population. The mtDNA variants are indicated along the branches of the tree.

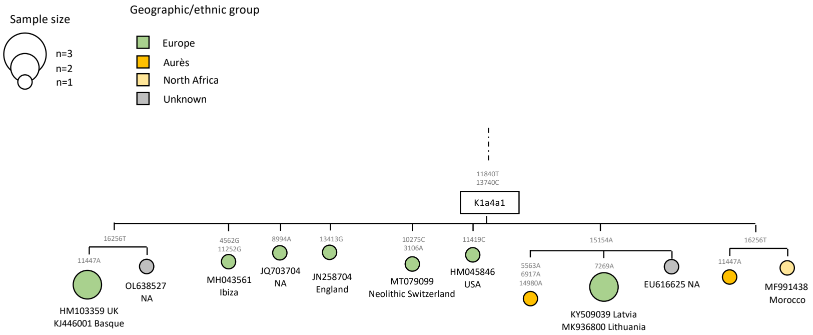

Figure 10 Maximum parsimony tree of the K1a4a1 haplogroup mitogenomes in the Aurès population. The mtDNA variants are indicated along the branches of the tree.

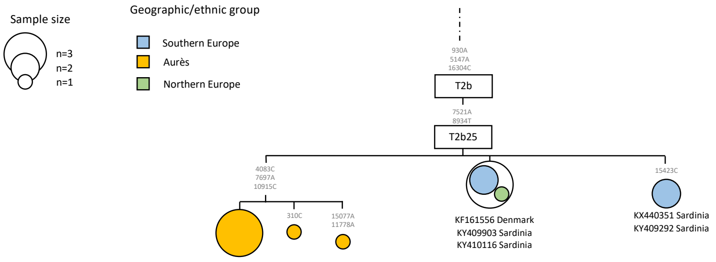

Figure 11 Maximum parsimony tree of the T2b25 haplogroup mitogenomes in the Aurès population. The mtDNA variants are indicated along the branches of the tree.

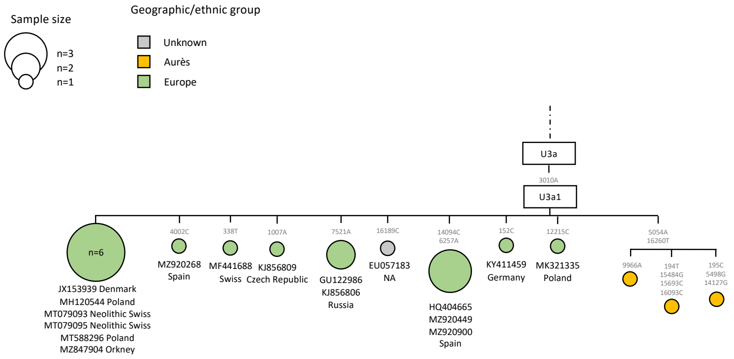

Figure 12 Maximum parsimony tree of the U3a1 haplogroup mitogenomes in the Aurès population. The mtDNA variants are indicated along the branches of the tree.

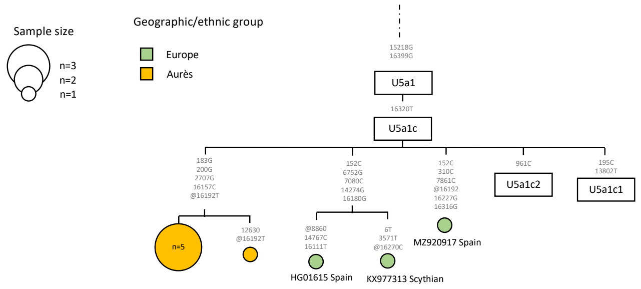

Figure 13 Maximum parsimony tree of the U5a1c haplogroup mitogenomes in the Aurès population. The mtDNA variants are indicated along the branches of the tree.

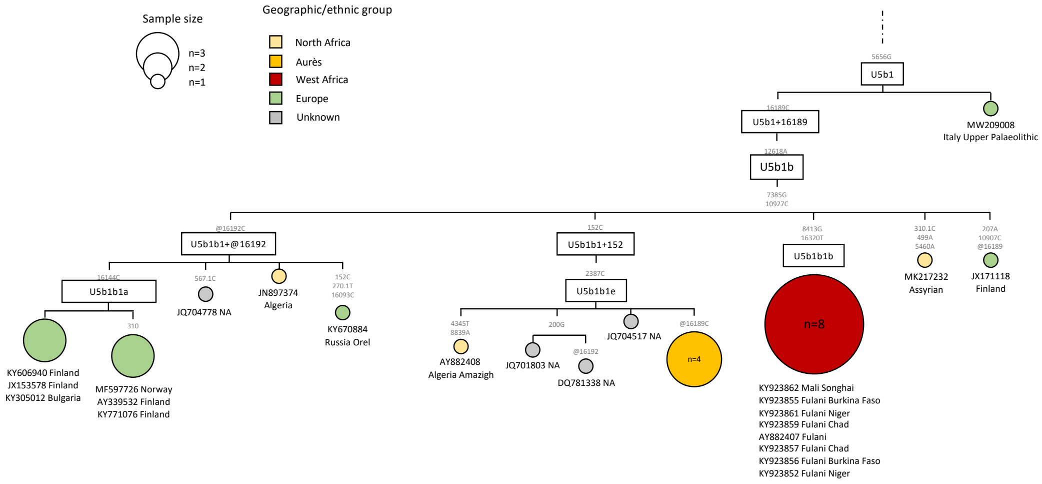

Figure 14 Maximum parsimony tree of the U5b1b1e haplogroup mitogenomes in the Aurès population. The mtDNA variants are indicated along the branches of the tree.

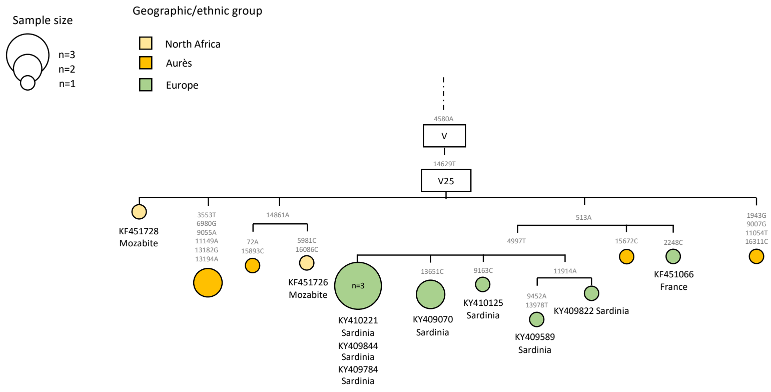

Figure 15 Maximum parsimony tree of the V25 haplogroup mitogenomes in the Aurès population. The mtDNA variants are indicated along the branches of the tree.

Middle Eastern lineages in the Aurès population

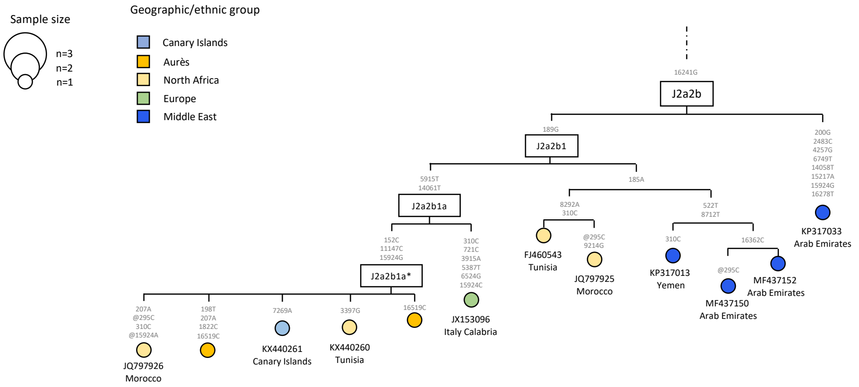

Figure 16 Maximum parsimony tree of the J2a2b1a haplogroup mitogenomes in the Aurès population. The mtDNA variants are indicated along the branches of the tree.*

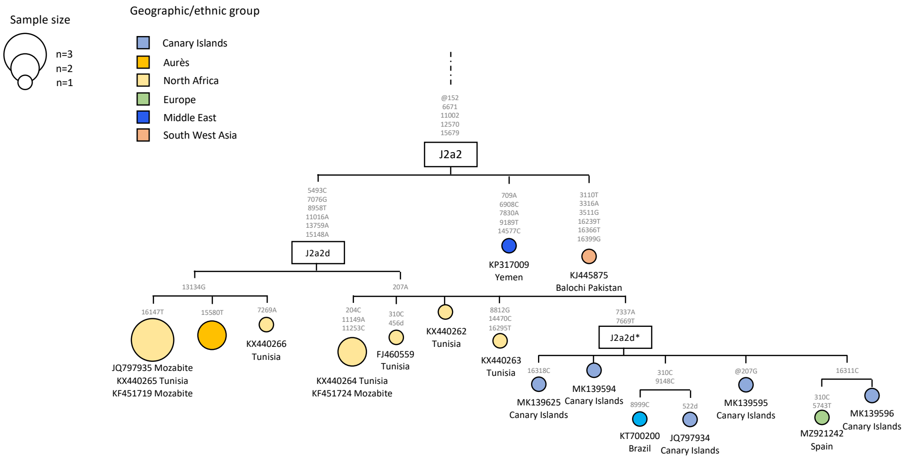

Figure 17 Maximum parsimony tree of the J2a2d haplogroup mitogenomes in the Aurès population. The mtDNA variants are indicated along the branches of the tree.

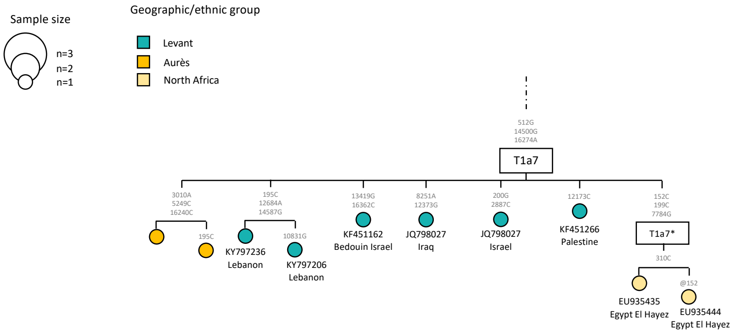

Figure 18 Maximum parsimony tree of the T1a7 haplogroup mitogenomes in the Aurès population. The mtDNA variants are indicated along the branches of the tree.

Sub-Saharan lineages in the Aurès population

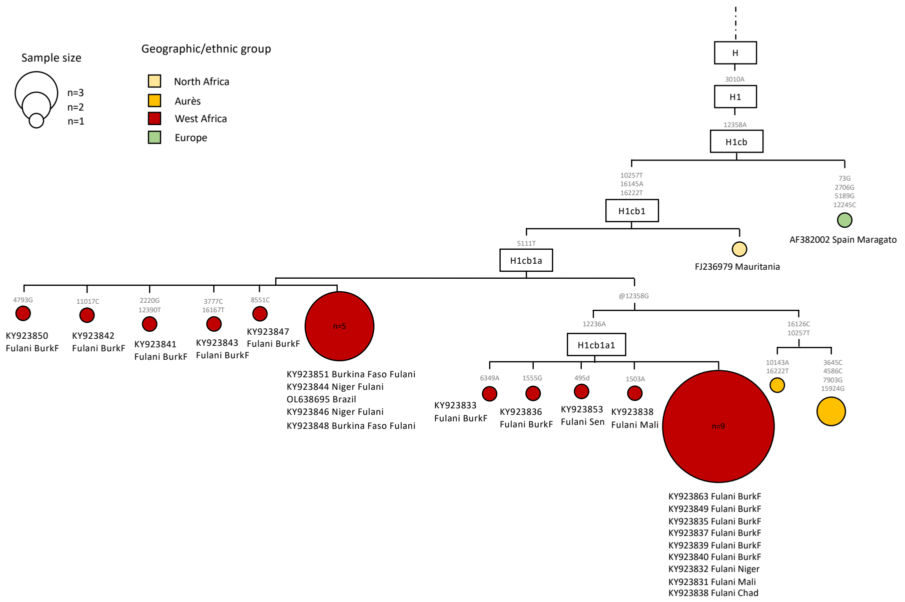

Figure 19 Maximum parsimony tree of the H1cb1a haplogroup mitogenomes in the Aurès population. The mtDNA variants are indicated along the branches of the tree.

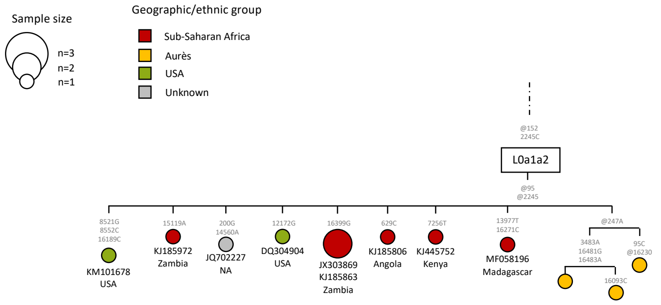

Figure 20 Maximum parsimony tree of the L0a1a2 haplogroup mitogenomes in the Aurès population. The mtDNA variants are indicated along the branches of the tree.

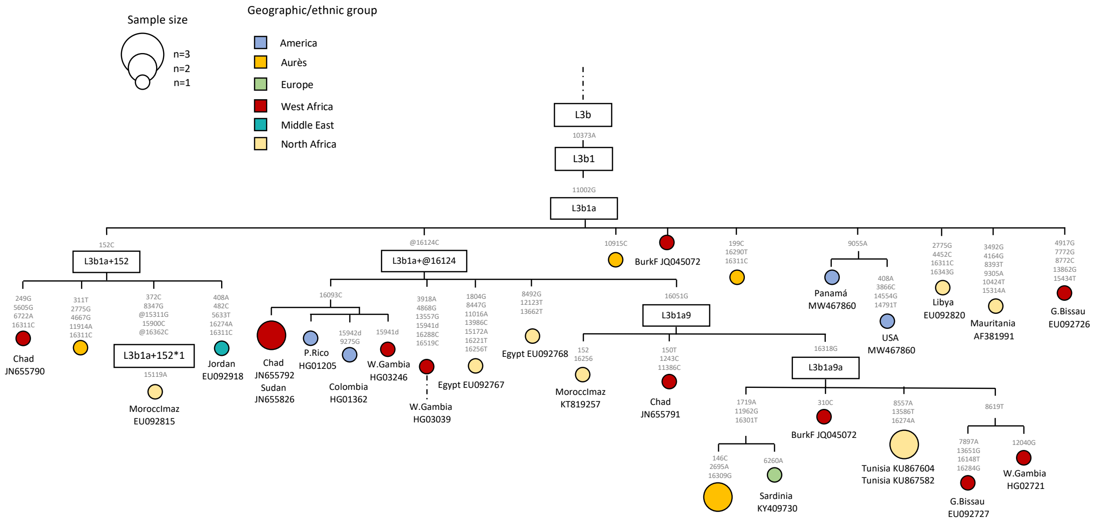

Figure 21 Maximum parsimony tree of the L3b1a9a haplogroup mitogenomes in the Aurès population. The mtDNA variants are indicated along the branches of the tree.

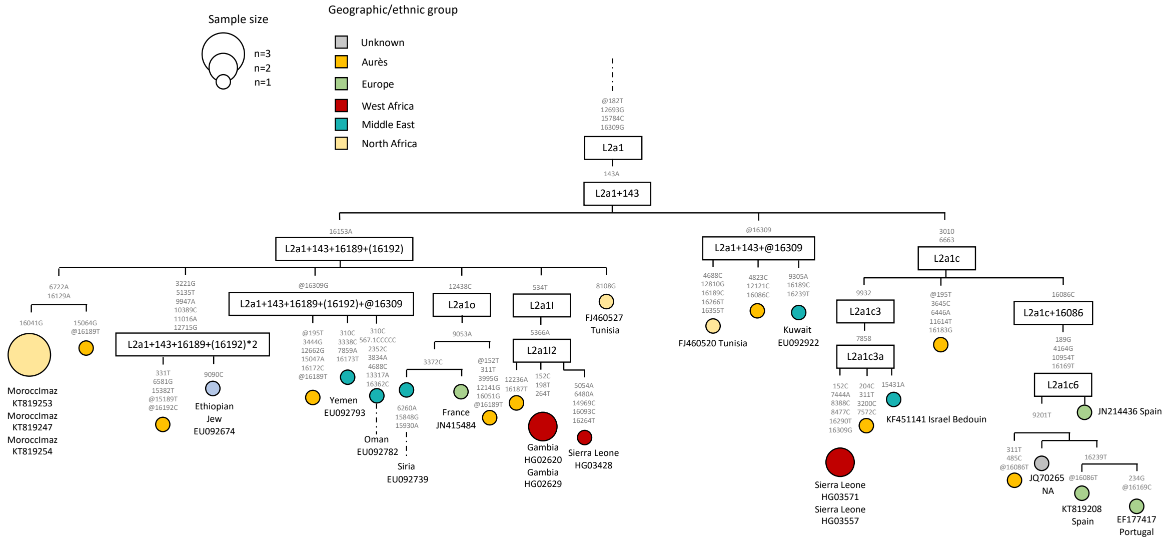

Figure 22 Maximum parsimony tree of the L2a1c6 and L2a1+143+16189+(16192) haplogroup mitogenomes in the Aurès population. The mtDNA variants are indicated along the branches of the tree.

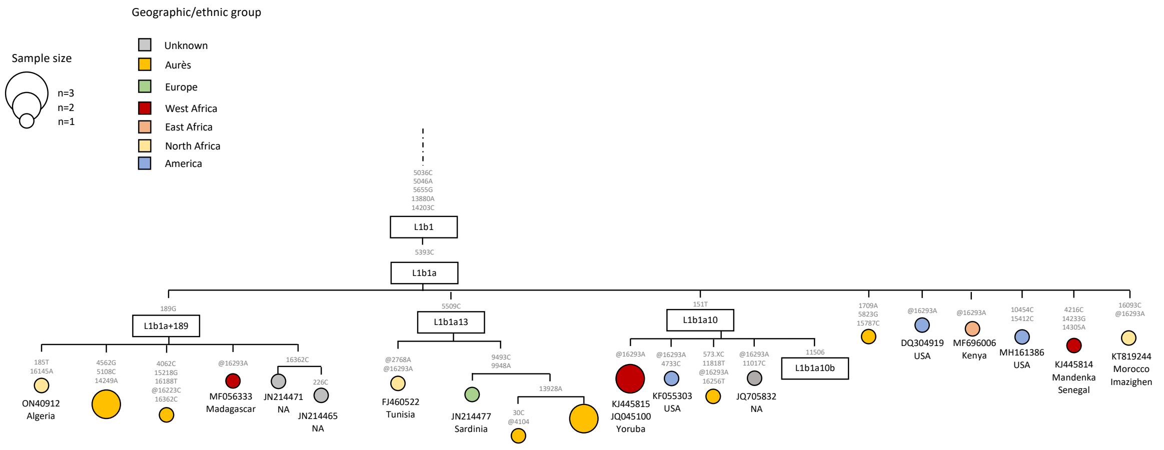

Figure 23 Maximum parsimony tree of the L1b1a haplogroup mitogenomes in the Aurès population. The mtDNA variants are indicated along the branches of the tree.

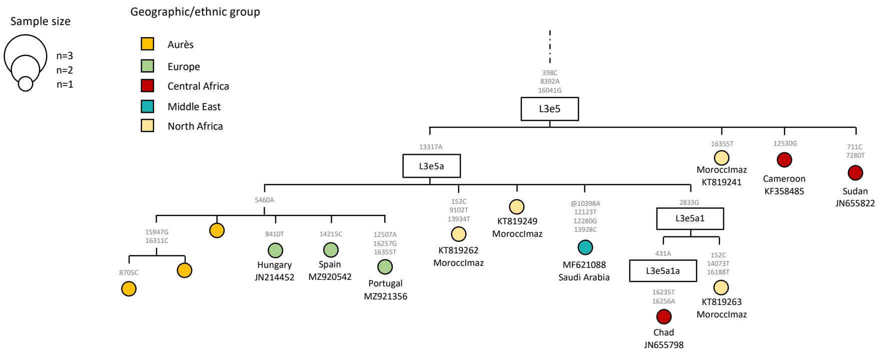

Figure 24 Maximum parsimony tree of the L3e5a haplogroup mitogenomes in the Aurès population. The mtDNA variants are indicated along the branches of the tree.

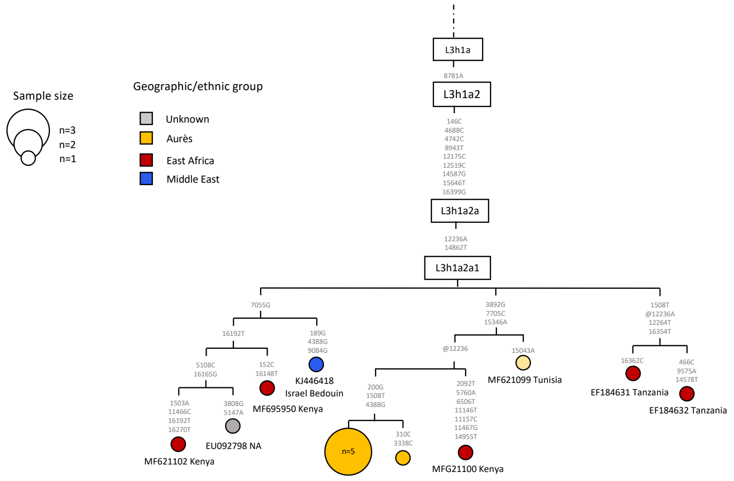

Figure 25 Maximum parsimony tree of the L3h1a2a1 haplogroup mitogenomes in the Aurès population. The mtDNA variants are indicated along the branches of the tree.

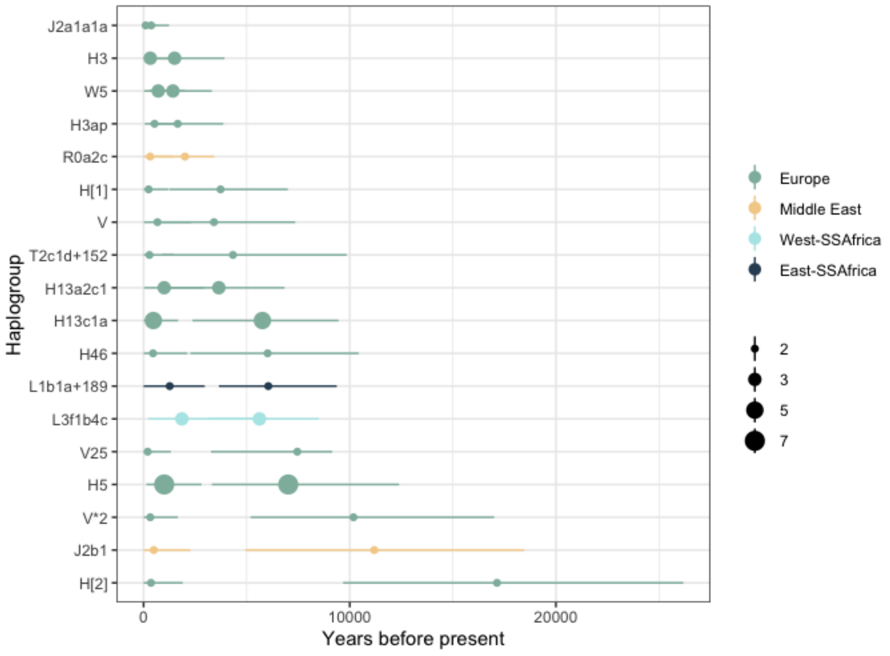

Figure 26 Inferred time divergences between North African identical sequences (leftmost point) and the closest non-North African sequence (rightmost point). Colours refer to the most likely geographic origin of these lineages, point ranges represent the 95% confidence intervals and the point size refers to the number of sequences within each lineage.

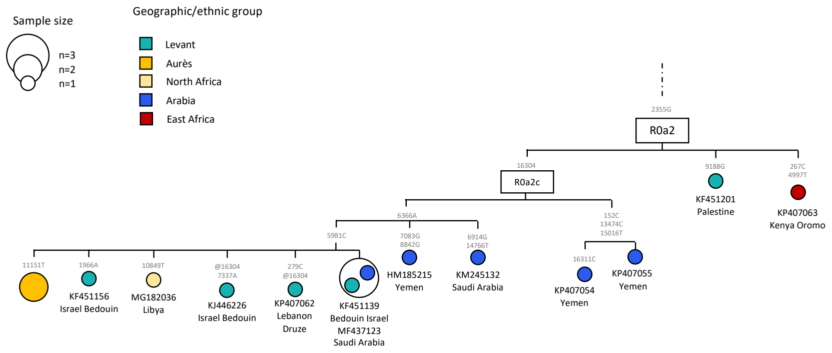

Figure 27 Maximum parsimony tree of the R0a2c haplogroup mitogenomes in the Aurès population. The mtDNA variants are indicated along the branches of the tree.

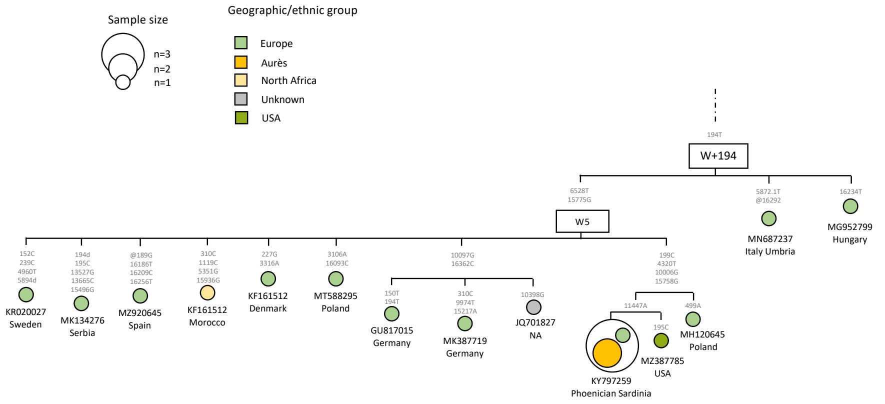

Figure 28 Maximum parsimony tree of the W5 haplogroup mitogenomes in the Aurès population. The mtDNA variants are indicated along the branches of the tree.

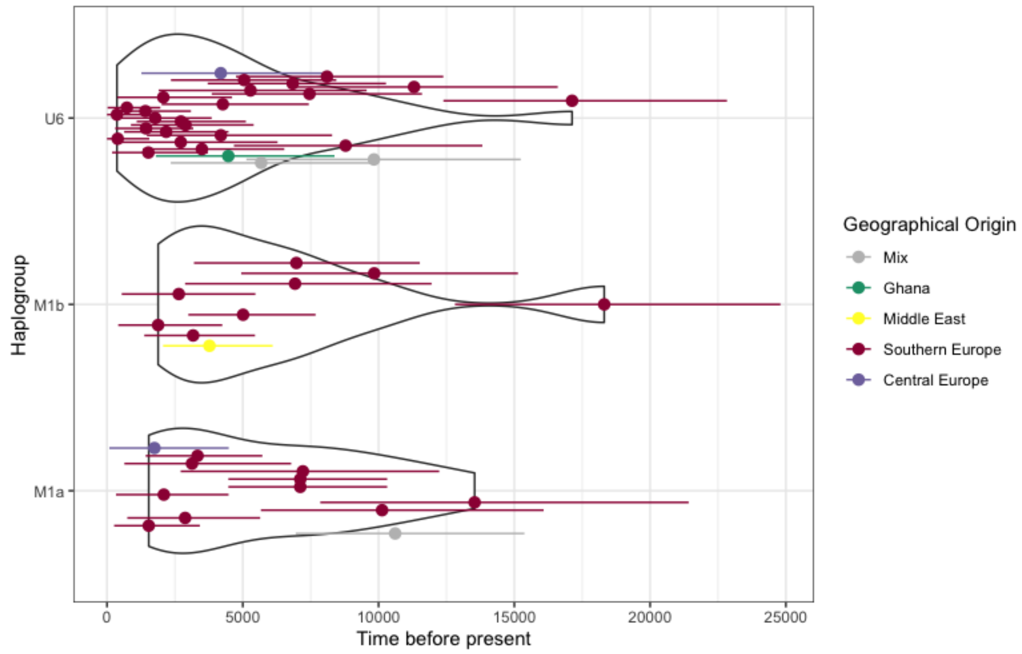

Figure 29 Time divergence between non-North African and closest North African sequences for M1 and U6 haplogroups. The point ranges represent the 95% confidence intervals and the geographic origin of the non-North African sequences are indicated by colors.

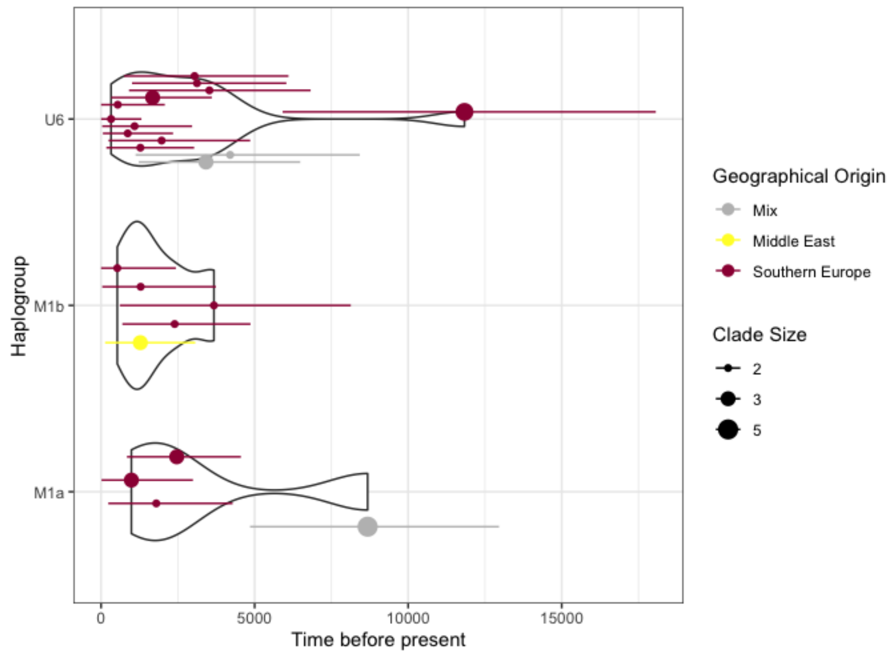

Figure 30 Internal time divergence for the European specific clades within U6 and M1 haplogroups. The point ranges represent the 95% confidence intervals, the point size indicate the size of the clades and the geographic origin of the clades are indicated by colors.
